# Whole-Exome Sequencing Analysis of Human Semen Quality in Russian Multiethnic Population

**DOI:** 10.3389/fgene.2021.662846

**Published:** 2021-06-11

**Authors:** Semyon Kolmykov, Gennady Vasiliev, Ludmila Osadchuk, Maxim Kleschev, Alexander Osadchuk

**Affiliations:** ^1^Institute of Cytology and Genetics, Siberian Branch of Russian Academy of Sciences, Novosibirsk, Russia; ^2^Department of Computational Biology, Sirius University of Science and Technology, Sochi, Russia

**Keywords:** whole-exome sequencing, association analysis, semen quality, pathozoospermia, normospermia, Slavs, Buryats, Yakuts

## Abstract

The global trend toward the reduction of human spermatogenic function observed in many countries, including Russia, raised the problem of extensive screening and monitoring of male fertility and elucidation of its genetic and ethnic mechanisms. Recently, whole-exome sequencing (WES) was developed as a powerful tool for genetic analysis of complex traits. We present here the first Russian WES study for identification of new genes associated with semen quality. The experimental 3 × 2 design of the WES study was based on the analysis of 157 samples including three ethnic groups—Slavs (59), Buryats (*n* = 49), and Yakuts (*n* = 49), and two different semen quality groups—pathozoospermia (*n* = 95) and normospermia (*n* = 62). Additionally, our WES study group was negative for complete AZF microdeletions of the Y-chromosome. The normospermia group included men with normal sperm parameters in accordance with the WHO-recommended reference limit. The pathozoospermia group included men with impaired semen quality, namely, with any combined parameters of sperm concentration <15 × 10^6^/ml, and/or progressive motility <32%, and/or normal morphology <4%. The WES was performed for all 157 samples. Subsequent calling and filtering of variants were carried out according to the GATK Best Practices recommendations. On the genotyping stage, the samples were combined into four cohorts: three sets corresponded to three ethnic groups, and the fourth set contained all the 157 whole-exome samples. Association of the obtained polymorphisms with semen quality parameters was investigated using the χ2 test. To prioritize the obtained variants associated with pathozoospermia, their effects were determined using Ensembl Variant Effect Predictor. Moreover, polymorphisms located in genes expressed in the testis were revealed based on the genomic annotation. As a result, the nine potential SNP markers rs6971091, rs557806, rs610308, rs556052, rs1289658, rs278981, rs1129172, rs12268007, and rs17228441 were selected for subsequent verification on our previously collected population sample (about 1,500 males). The selected variants located in seven genes *FAM71F1*, *PPP1R15A*, *TRIM45*, *PRAME*, *RBM47*, *WDFY4*, and *FSIP2* that are expressed in the testis and play an important role in cell proliferation, meiosis, and apoptosis.

## Introduction

Infertility is a worldwide problem, and 10–15% of married couples are unable to have children (Sharlip et al., 2002). Infertility due to male factor ranged from 20 to 70%, and the percentage of infertile men ranged from 2.5 to 12% in different populations. Infertility rate was the highest in Africa and Central/Eastern Europe. Additionally, according to the variety of sources, the infertility rate of males in North America, Australia, and Central and Eastern Europe varied from 4.5 to 6%, 9, and 8 to 12%, respectively (Agarwal et al., 2015). In the Russian Federation for the 2000–2018 period, there was an increase in the total number of infertile men (an increase of 2.1 times), as well as an increase of 82% in men with primary male infertility. In all Federal Districts except the Far East Federal District, the urological disease incidence has been increased (Lebedev et al., 2019).

Over the past three decades, a number of studies and comprehensive meta-analyses showed a time-related decrease of semen quality as well as an increase of male infertility and incidence of some diseases associated with the male reproductive system (Giwercman et al., 1993; Swan et al., 2000; Rolland et al., 2013; Kumar and Singh, 2015; Skakkebaek et al., 2016; Levine et al., 2017; Sengupta et al., 2017; Wang et al., 2017; Mishra et al., 2018; Mínguez-Alarcón et al., 2018; Siqueira et al., 2020). In some populations, semen quality has reached a level where a significant proportion of men of reproductive age were at risk of subfertility or infertility. Moreover, it has been shown that impaired semen quality or male infertility may be associated with shorter life expectancy and increased morbidity (Eisenberg et al., 2014; Latif et al., 2017; Glazer et al., 2019). In addition, numerous studies have identified considerable regional differences in semen quality and the prevalence of certain diseases associated with urogenital disorders (Jørgensen et al., 2001, 2002, 2006; Punab et al., 2002; Swan et al., 2003; López-Teijón et al., 2008; Paasch et al., 2008; Fernandez et al., 2012; Halling et al., 2013; Redmon et al., 2013; Erenpreiss et al., 2017; Osadchuk et al., 2020). The reasons of temporal or geographic differences in semen quality remain poorly understood; however, different climatic conditions, environmental toxicants, lifestyle, and genetic background are considered as important contributors to male reproductive health (Skakkebaek et al., 2016).

In the last decade, the extraordinary progress in the development of next-generation sequencing (NGS) technologies have stimulated the development of several commercial platforms suitable for the effective molecular genetic analysis of numerous diseases associated with male infertility and subfertility. Currently, whole-exome analysis (WES) is the most optimal approach for solving a variety of biomedical problems. The human whole exome includes about 180,000 exons of all protein-coding genes, which is about 1% of the whole genome, or about 30 million bp. Approximately 85% of the known mutations which cause various human diseases, occur in this part of the genome. Currently, the usage of WES in studying of the genetic causes of impaired spermatogenesis has a relatively short history of 4–6 years.

There is a problem in identifying genetic variants of idiopathic infertility in men. For example, the multigenic nature of non-obstructive azoospermia requires a large population to identify mutations that lead to this condition. On the contrary, new genes are more likely to be detected in the setting of high consanguinity by using NGS technologies (Fakhro et al., 2018). A recent review analyzed 23 studies that used a WES (Robay et al., 2018). In these studies, 28 genes were found, whose mutations led to non-obstructive azoospermia; 18 of them caused quantitative changes in sperm parameters, including five mutations which led to defects of sperm morphology and five which led to a decrease in sperm motility. For the mentioned studies, the analysis of consanguineous families was mainly used; the number of exon sequencing ranged from 1 to 59, with an average of 14 exomes. A unique study was conducted on 186 exomes (Fakhro et al., 2018), which included 37 men from eight consanguineous families, as well as 149 men, including 75 unrelated men with non-obstructive azoospermia and 74 unrelated men with proven fertility. Analysis of the obtained WES data made it possible to identify new rare recessive variants of the *CCDC155*, *NANOS2*, *SPO11*, *TEX14*, and *WNK3* genes associated with non-obstructive azoospermia, which were expressed exclusively in the testes and were not found in the fertile control. Another group of authors managed to identify three novel causative mutations of azoospermia in three genes: *MIO*, *TEX14*, and *DNAH6* in brothers from three families (Gershoni et al., 2017). These genes were associated with different meiotic processes: meiotic crossovers, daughter cell abscission, and possibly rapid prophase movements. A non-stop mutation of the *MAGEB4* gene localized on the X-chromosome was identified in a related family from Turkey in which the brothers suffered from infertility caused by azoospermia (Okutman et al., 2017).

Using WES, molecular aspects of infertility associated with defects of normal sperm morphology were demonstrated. In particular, a homologous mutation (c.G2783A, p.G928D) in the *BRAF* gene was identified in a patient from a consanguineous family (Li et al., 2017). The gene product, BRDT, was a testis-specific protein that was considered an important drug target for male contraception. In addition, WES analysis identified a homozygous deletion within the *TSGA10* gene (c.211delG; p.A71Hfs^∗^12) which resulted in the production of truncated TSGA10 protein. TSGA10 is a testis-specific protein that localized in the midpiece of the normal spermatozoa (Sha Y. W. et al., 2018). The successful WES application in searching for new genes, whose mutations cause the loss of sperm motility, has been described, for example, in the syndrome of multiple morphological abnormalities of sperm tail, leading to infertility (Amiri-Yekta et al., 2016; Kherraf et al., 2018; Martinez et al., 2018). WES analysis of 78 infertile men with multiple morphological abnormalities of the sperm flagella (MMAF) phenotype permitted the identification of four homozygous mutations in the fibrous sheath (FS)-interacting protein 2 (*FSIP2*) gene in four unrelated individuals (Martinez et al., 2018). Based on WES analysis, biallelic mutations of *CFAP43* and *CFAP44* genes in four out of 30 Chinese men were associated with multiple tail abnormalities and decreased sperm motility (Tang et al., 2017). The study of the pathogenesis of acephalic sperm syndrome as a rare form of teratozoospermia was carried out by WES analysis and revealed 10 biallelic mutant variants of the SUN5 gene, which accounted for 47.06% of cases of genetic defects in patients with this pathology (Zhu et al., 2016). Further WES analysis identified a homozygous nonsense mutation of the *PMFBP1* gene, which was expressed only in the testes (Zhu et al., 2018).

Given to the small number of whole-exome studies investigating molecular genetic causes of infertility, the following conclusions can be drawn: (1) the purpose of the conducted studies, as a rule, was diagnostic; (2) the WES analysis was primarily used on a small cohort of patients with clearly expressed qualitative reproductive defects; (3) in the near future, we should expect a greater research flow in this area, due to the cheaper methodical base and the development of new bioinformatic approaches in analyzing the WES results.

The aim of the study was to identify novel genetic variations that would affect the semen quality parameters. To achieve this purpose, WES analysis was used. The advantage of this technology is the identification of the new associations between gene mutations and clinical markers of sperm quality (sperm concentration, motility, and morphology). We used an experimental 3 × 2 design for our WES study based on the analysis of 157 samples from men of three ethnic groups—Slavs, Buryats, and Yakuts, and two groups of men, contrasting in semen quality—pathozoospermia and normospermia. Additionally, our WES study group was negative for complete AZF microdeletions of the Y-chromosome, which are the established cause of azoospermia. Thus, our WES approach was significantly different from the ones described above. Essentially, our analytical design was three-dimensional, involving three factors—genotype, semen quality, and ethnicity. The developed approach made it possible to distinguish two types of genetic loci, one of which had additive effects characterizing the entire study population, the other which was related only to certain ethnic groups.

## Materials and Methods

### Study Population

The total population sample of male volunteers (*n* ≈ 1900) was selected in a wide Eurasian area (about 5,500 km), including six cities of the Russian Federation and the Republic of Belarus: Arkhangelsk, Novosibirsk, Kemerovo, Ulan-Ude, Yakutsk, and Minsk. The cities of Minsk, Novosibirsk, and Kemerovo have a predominantly Slavic population (approximately 95%). Ulan-Ude is inhabited by 32% of Buryats, and Yakutsk is inhabited by 43% of Yakuts. At all cities, the study design was the same, and we used a standardized recruitment protocol, which was earlier written elsewhere in more detail (Osadchuk et al., 2020). Briefly, inclusion criteria for participation in the study were absence of acute general diseases or chronic illness in an acute phase, and genial tract infections. Each participant was warned about the necessity of sexual abstinence for 2–3 days before the examination. All participants gave informed consent to participation in the examination. Each participant filled in a standardized questionnaire which included information about age, place of birth, self-identified nationality of the participant, his parents and grandparents, family status, alcohol consumption, tobacco smoking, profession, and previous urological diseases. All study subjects were volunteers and did not receive any financial compensation. The ethics committee of the Federal Research Center “Institute of Cytology and Genetics,” the Siberian Branch of the Russian Academy of Sciences, approved the study.

### Physical Examination, Blood and Sperm Collection, Semen Analysis, and DNA Extraction

Physical examination, blood and sperm collection, and semen analysis were described in detail earlier (Osadchuk et al., 2020, 2021). Briefly, all participants were examined by an experienced andrologist, who diagnosed urogenital disorders. Body weight (kg), height, waist and hip circumference (cm), and body mass index (BMI, kg/m2) were determined. Testicular volume (ml) was estimated using a Prader orchidometer and was presented as bitesticular volume (paired testicular volume). Each participant provided both blood from the cubital vein and semen sample by masturbation. The serum samples were stored at –400 C until the analysis. Genomic DNA was extracted from peripheral blood samples using the standard phenyl-chloroform method.

The semen samples were analyzed for semen volume (ml), sperm concentration (× 106/ml), and morphology (percentage) according to the WHO laboratory manual for the examination and processing of human semen [World Health Organization (WHO), 2010], but sperm progressive motility was determined by using the automatic sperm analyzer SFA-500 (Biola, Russia). Total sperm count was then calculated by multiplying the individual’s sperm concentration by the ejaculate volume.

To assess sperm morphology, ejaculate smears were prepared, fixed by methanol, and stained by using commercially available kits Diff-Quick (Abris plus, Russia) according to the manufacturer’s manual. Two hundred spermatozoa were examined for morphology with an optical microscope (Axio Scope A1, Carl Zeiss, Germany) at ×1,000 magnification with oil immersion and the sperm anomalies were listed according to the WHO guidelines [World Health Organization (WHO), 2010], and the percentage of sperm scored as morphologically normal (%) was used.

### Preselected Group of Men for Whole-Exome Sequencing Analysis

For the WES analysis, 157 participants were selected from the whole study sample. According to the 3 × 2 design of our WES study, the sample was divided into three ethnic groups—Slavs, Buryats, and Yakuts, and two groups contrasting in sperm quality—pathozoospermia and normospermia (Supplementary File 1). The pathozoospermia group included men with sperm concentration <15 × 10^6^/ml, and/or progressive motility <32%, and/or normal morphology <4%, whereas the normozoospermia group included men with these semen parameters equal or higher than normal reference values [World Health Organization (WHO), 2010]. The distribution of the preselected group of 157 men by ethnicity and sperm quality is shown in Table 1. In general, 60.5% of the participants were characterized by pathozoospermia, the remaining 39.5%—normospermia. The preselected sample was aligned by ethnic composition and did not contain complete deletions of AZFc and AZFa regions of the Y-chromosome associated with azoospermia.

**TABLE 1:** emen and ethnic characteristics of men in the preselected sample prepared for the whole exome sequencing.

The structure of semen quality	Ethnic groups				Frequency to the total sum,%
	Slavs	Buryats	Yakuts	Sum	
Normospermia—NNN	23	20	19	62	39.5%
Az	13	1	3	17	10.8%
OAT	16	14	20	50	31.8%
OA	6	1	2	9	5.7%
OAN	1	13	5	19	12.1%
Sum of pathozoospermia	36	29	30	95	60.5%
Total sum	59	49	49	157	
Pathozoospermia,%	61.0%	59.2%	61.2%		

*O, oligozoospermia; A, asthenozoospermia; T, teratozoospermia; N, Norma; Az, azoospermia.*

Almost 11% of the participants in the preselected sample were characterized by azoospermia. Three indicators of spermatogenic failure characterized the remaining participants in the pathozoospermia group: oligo-, astheno-, and teratozoospermia. The group of oligoasthenoteratozoospermia (31.8%) was the largest. Because of the very low sperm concentration in men with oligoasthenozoospermia (5.7%), it was not correct to estimate sperm morphology. The group of oligoasthenozoospermia with normal sperm morphology (12.1%) was the last in the list of different forms of pathozoospermia.

### Whole-Exome Sequencing Procedure

Genome libraries were prepared using the Illumina TrueSeq DNA Library Prep for Enrichment according to the manufacturer’s manual with minor modifications. DNA was fragmented on a Covaris M220 device with parameters optimized for a maximum of fragments in the range of 150–200 bp. One hundred nanograms of fragmented DNA were used to create genomic libraries with average insertion sizes 150–200 bp. Size selection protocol was modified: 86 μl SPB was added to end repair mixture; 125 μl SPB was added to the supernatant from the previous step. The further procedure was carried out according to the manufacturer’s manual, and amplification of libraries was carried out in 9 cycles. The quality and molarity of the resulting libraries were determined using a Bioanalyzer BA2100. One hundred fifty-seven high-quality libraries were combined in 10 groups for hybridization (6 × 16 and 3 × 15). Final exome libraries were obtained using Illumina XGen Exome Research Panel v1.0 according to the manufacturer’s manual. One hundred twenty-five nanograms of each library was used (4plex calculation); sample concentration was performed by adding 1.8 volumes of AMPure XP. Hybridization was being carried out for 5 h. Each set of exome libraries (16 or 15) was sequenced on a NextSeq550 sequencer using the NextSeq 550 High Output v2Kit 150 cycles (Illumina, United States). To determine AR gene full CAG repeat length, we had to use a single direct 150-bp read.

### Whole-Exome Data Processing, Variant Calling, and Quality Control

The reads were mapped to the human reference genome (GRCh38. p13; GCA_000001405.15) using the BWA-MEM algorithm (Li, 2013). To remove duplicate reads, Picard MarkDuplicates was used. The mean depth of sequencing coverage for the exome data was 47.5 with coverage >20 × for 82% of target bases.

**FIGURE 1:**
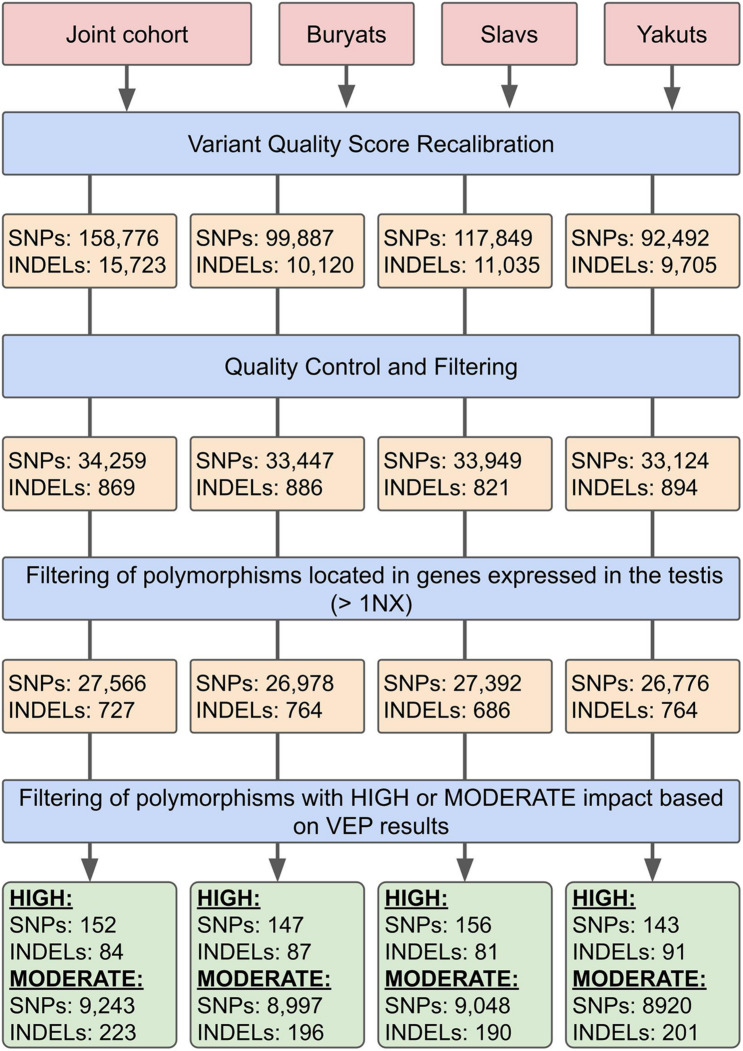
Whole-exome genotype data filtering pipeline.

Variant calling and filtering were performed according to the GATK Best Practices recommendations. SNP identification, as well as insertions and deletions (INDELs), was identified using HaplotypeCaller and GenotypeGVCFs from Genome Analysis Toolkit (GATK) v4.1.4.1 (Poplin et al., 2017). On the genotyping stage, the samples were combined into four cohorts: three sets corresponded to three ethnic groups, and the fourth set contained all 157 whole-exome samples (Figure 1).

For the subsequent filtering of the obtained sets of mutations at the first stage, the Variant Quality Score Recalibration (VQSR) algorithm was used. The algorithm uses a machine learning approach to determine the threshold values of various parameters describing the quality of the considered sets of mutations. Changes in the number of polymorphisms at different stages of filtration are shown in Figure 1. HapMap v3.3, dbSNP 146 (Sherry et al., 2001) and the 1000 Genomes data (1000 Genomes Project Consortium, 2015) were used as training data. On the second stage of filtering the studied sets of SNPs and INDELs, the considered data were converted into the PLINK BED format (Chang et al., 2015). Poorly presented (<2% of the number of samples) polymorphisms were filtered out, as well as the ones deviating from the Hardy–Weinberg equilibrium with a threshold of 10^–6^ in controls. Subsequently, polymorphisms with a Minor Allele Frequency <10% were removed. The heterozygosity rate of the samples participating in the study was also analyzed. According to the results, none of the samples deviated ±3 SD from the heterozygosity rate mean. The quality control and association analyses are partly based on the analysis described by Marees et al. (2018).

### Annotation

The obtained sets of polymorphisms were annotated using Annovar (dbSNP146) (Wang et al., 2010). To prioritize the variations associated with pathozoospermia, their effects were determined using Ensembl Variant Effect Predictor (McLaren et al., 2016). Moreover, polymorphisms located in genes expressed in the tissues of the male reproductive system were revealed based on genomic annotation. Gene expression data were obtained from the Human Protein Atlas database (Uhlén et al., 2015).

Based on these expression data, to each polymorphism harboring gene were assigned both expression in the testes and expression levels in individual cell types: spermatocytes, spermatogonia, and early and late spermatids. To check whether the relationship of the obtained polymorphisms with some complex traits was shown in previous studies, ClinVar (Landrum et al., 2014) and PhenoScanner (v2) (Staley et al., 2016) databases were used. Also, the DisGeNET database was used to identify genes associated with male infertility (Piñero et al., 2016). For this purpose, genes were selected for which an association with various pathological phenotypes was shown. Thus, a list of 832 genes associated with male infertility was compiled.

### Statistical Analysis

To assess the effects of continuous variables (age, frequency of alcohol, and smoking) that may influence sperm quality parameters, we performed an analysis of covariance (ANCOVA) using the STATISTICA 8.0 software package. It turned out that none of the above covariates had a significant effect on all four parameters of sperm quality (total number, concentration, sperm motility in the ejaculate, and the ratio of sperm with normal morphology).

The association of the obtained polymorphisms with spermatogenic parameters in the joint cohort was investigated using the χ^2^ test (PLINK –assoc). Since the χ^2^ test does not provide an opportunity to include additional covariances in the analysis, an additional logistic regression method was used to search for the associations (PLINK –assoc –logistic). The values of external factors, presumably influencing the efficiency of spermatogenesis, were used as covariances: age, the number of cigarettes smoked, and the amount of alcohol consumed per week. Also, in the case of a joint sample, 10 principal components were used, obtained at the stage of stratification analysis and describing the internal population structure of the sample under study. The list of polymorphisms was tested for pairwise independence of the spermatogenesis pattern, genotype, and ethnicity using analysis (Zar, 1984) of three-dimensional 3 × 3 × 2 contingency tables (3 genotypes for autosomal SNP marker × 3 ethnic groups × 2 ranks of sperm quality). A statistical analysis of the obtained data was performed using the statistical package ‘‘STATISTICA’’ (version 8.0) and R statistical software^1^.

The influence of rare variants on semen quality was analyzed using sequence kernel association tests implemented in the SKAT software package (Wu et al., 2011).

## Results

**FIGURE 2:**
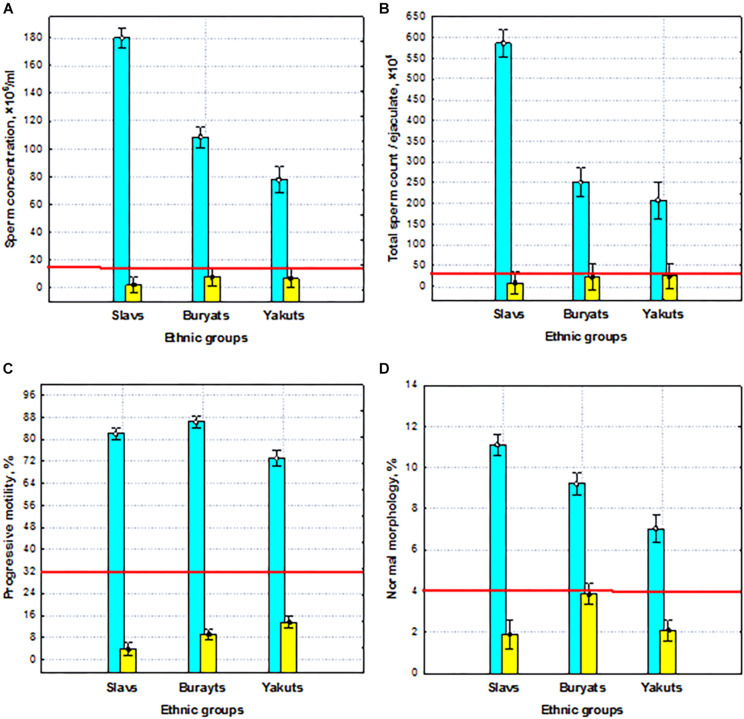
Sperm quality (normospermia and pathozoospermia) and ethnic effects on semen quality parameters. **(A)** Sperm concentration, **(B)** total sperm count, **(C)** progressive sperm motility, and **(D)** normal sperm morphology in the preselected sample. Blue columns—normospermia, yellow columns—pathozoospermia. The red lines indicate the WHO reference limits for different normal semen parameters [World Health Organization (WHO), 2010].

Our experimental 3 × 2 design allows us to detect common patterns of phenotypic and genetic variability of the male reproductive potential and to investigate its ethno-specific features. The results of two-way ANOVA revealed highly significant (*p* < 0.001) differences between the groups with normospermia and pathozoospermia, as well as between the three ethnic groups in terms of semen quality, including the total sperm count, concentration, motility, and morphology (Figure 2). At the same time, highly significant interactions (*p* < 0.001) between two studied factors for all sperm parameters indicated that the features of ethnic differences depend on sperm quality—normospermia and pathozoospermia.

Thus, the preselected sample was characterized by extraordinary differences in all sperm quality indicators between normal and pathozoospermia groups and between ethnic groups, which are a reliable basis for identifying genes responsible for spermatogenic failure by WES.

### Analysis of Population Stratification

**FIGURE 3:**
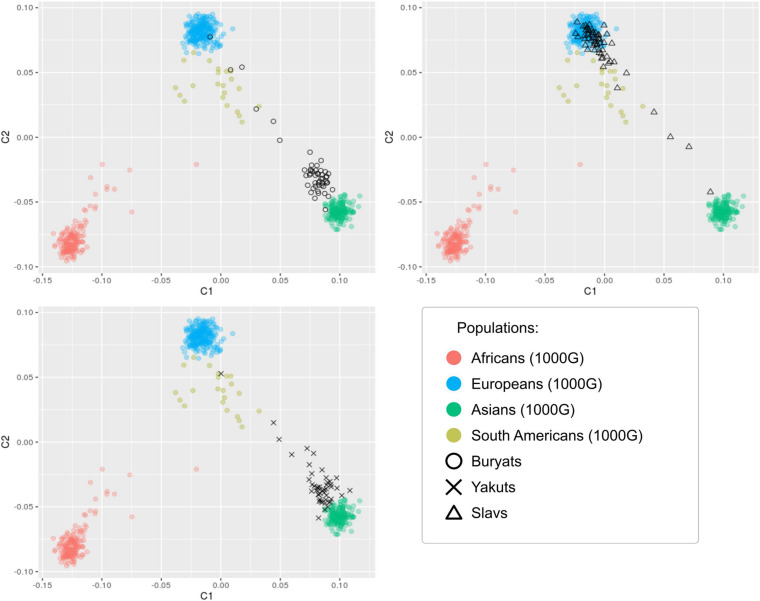
Visualization of the first two components of the results of the multidimensional scaling analysis of 157 samples in the context of other populations.

The heterogeneity of the studied sets of genotypes was assessed (Figure 3). Genomic control inflation factors λ were evaluated to be 1.075, 1.012, and 1.019 for Buryats, Slavs, and Yakuts, respectively, which corresponds to a low degree of population stratification (Picard, 2021). The visualization of the first two components of the multidimensional scaling shows an explicit clustering of the studied data sets according to their ethnic groups. It was observed that the populations of Buryats and Yakuts are clustered separately from the reference data, while the Slavic population overlaps the European population. Similar cases have been shown in other WES studies of the Siberian populations (Fedorova et al., 2013; Cardona et al., 2014). Worth noting are a small number of samples lying outside the formed clusters. Samples that are ±2 SD from the sample mean of the first two principal component scores were removed from the ethnic-specific groups. These outliers were matched against the data description. According to the description of the samples, these samples have been shown to be metises. Thus, at the stage of stratification analysis, 3, 1, and 1 samples were excluded from further analysis from the ethnic-specific sets of polymorphisms: Buryats, Slavs, and Yakuts, respectively.

### Association Analysis

#### Common Variant Association Analysis for the Joint Set

**FIGURE 4:**
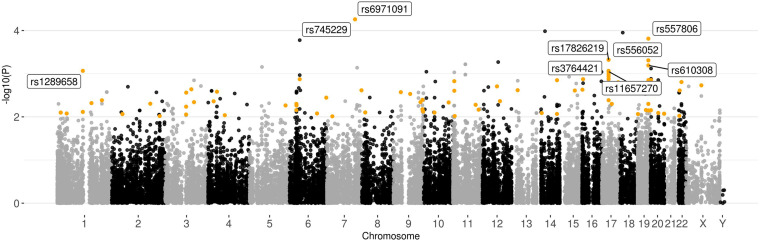
Graphical summary of the results of the association analysis of 157 samples. Plot of –log10(*p*-values) of the χ2 test. Polymorphisms with *p*-value < 10^–2^ and high or moderate impact based on the VEP results located in the genes expressed in the testis (>1NX) are highlighted.

At the genotyping stage after filtering based on quality analysis, 34,259 SNPs and 869 INDELS were identified for the joint set. To search for variations associated with impaired spermatogenesis in the joint sample, the obtained set of polymorphisms was analyzed using the χ2 test (Figure 4 and Supplementary File 2). As a result, two signals with *p*-value < 10^–4^ (rs745229 (*FAM71F1*) [*p*-value = 5.495 × 10^–5^, OR = 0.2992], rs6971091 (*FAM71F1*) [*p*-value = 5.495 × 10^–5^, OR = 0.2992]) and 23 signals with *p*-value in the range from 10^–3^ to 10^–4^ were identified However, the association analyses revealed no significant genetic associations surviving Bonferroni correction (0.05/35.495 = 1.4 × 10^–6^). In order not to overlook potential SNP markers due to statistical power limitations, lower significance thresholds (*p*-value < 10^–3^) were used.

To prioritize polymorphisms, the variations with high or moderate impact located in genes expressed in the tissues of the male reproductive system were filtered out from the obtained results. Only nine SNPs in the range *p*-value < 10^–3^ met these conditions: rs6971091, rs557806, rs17826219, rs556052, rs610308, rs1289658, rs3764421, rs11657270, and rs3816780. Among the synonymous polymorphisms with a high association with impaired spermatogenesis, rs9805910 (*TTC6*), and rs3741688 (*RAB21*) should be distinguished, since they are located in the genes that have increased expression in early and late spermatids.

Since the χ^2^ test is not able to take into account the effect of additional covariates in the analysis of associations, the logistic regression method was additionally used (Supplementary File 3 and Supplementary Figure 1 from Supplementary File 11). In addition to population stratification data, data on the amount of alcohol and cigarette consumption and age (Supplementary Files) were used as covariances. These results were compared with those obtained with the χ^2^ test. The associations of the previously determined SNPs—rs6971091, rs557806, rs1289658, and rs556052—were also confirmed using the logistic regression. Attention should be paid to two SNPs: rs55888197 and rs55860603, located in the *OR8U1* gene and demonstrating the highest level of the association. However, this case is most likely a consequence of the alignment of reads to the highly homologous *OR8U8* gene. Also, using the logistic regression model, associations with impaired spermatogenesis of two signals: rs1218825 (*MTIF3*) and rs1129172 (*PRAME*) were identified. The genes containing these polymorphisms show high levels of expression in the tissues of the male reproductive system.

The polymorphism pairwise independence of the spermatogenesis pattern, genotype, and ethnicity was tested (Table 2). Based on the results, polymorphisms whose predictive ability does not depend on ethnicity (rs6971091, rs557806, rs610308, rs556052, and rs1289658) were determined. It is worth noting that the polymorphisms located in the *ATAD5* gene failed the pairwise independence test.

**TABLE 2:** Results of χ2 tests for pairwise independence of the spermatogenesis pattern, genotype, and ethnicity of top polymorphisms with high or moderate impact based on VEP results in the joint set (*p*-value < 10^–3^) localized in the genes expressing in testis (>1NX).

SNP	Gene	There is no relationship between			
		Ethnicity and spermatogenesis pattern with genotype	Ethnicity, spermatogenesis pattern and genotype	Spermatogenesis pattern and ethnicity with genotype	Genotype and spermatogenesis pattern with ethnicity
rs6971091	*FAM71F1*	0.44294	**0.00426**	**0.01998**	**0.00129**
rs557806	*PPP1R15A*	0.0693	**0.00037**	**0.03918**	**0.0001**
rs610308	*PPP1R15A*	0.72918	0.11055	0.06213	0.05612
rs556052	*PPP1R15A*	0.80398	0.12313	0.05928	0.06312
rs1289658	*TRIM45*	0.05338	**0.00086**	**0.03311**	**0.00026**
rs3741688	*RAB21*	**0.03251**	**0.00284**	**0.01642**	**0.00086**
rs17826219	*ATAD5*	0.97571	0.10679	0.13231	**0.04887**
rs3764421	*ATAD5*	0.99979	0.29664	0.14557	0.17021

*Statistically significant results are boldfaced.*

In order to check whether the relationship of one of the obtained polymorphisms with some complex traits was shown in previous studies, we used the ClinVar and Phennoscanner databases. For rs557806, a significant association with neoplasms was shown. To obtain a list of genes associated with male infertility, the DisGeNET database was used. The complete list of polymorphisms detected for the joint sample was compared with the DisGeNET data. As a result, none of the genes harboring signals with *p*-value < 10^–3^ were found in the list with genes from DisGeNET (Supplementary File 1). Based on this comparison, it is possible to identify the gene in which the polymorphism with the most reliable association is localized—*TEX11* (rs4844247 [*p*-value = 1.861 × 10^–3^, OR = 6.076]).

#### Common Variant Association Analyses for the Ethnic-Specific Sets

**FIGURE 5:**
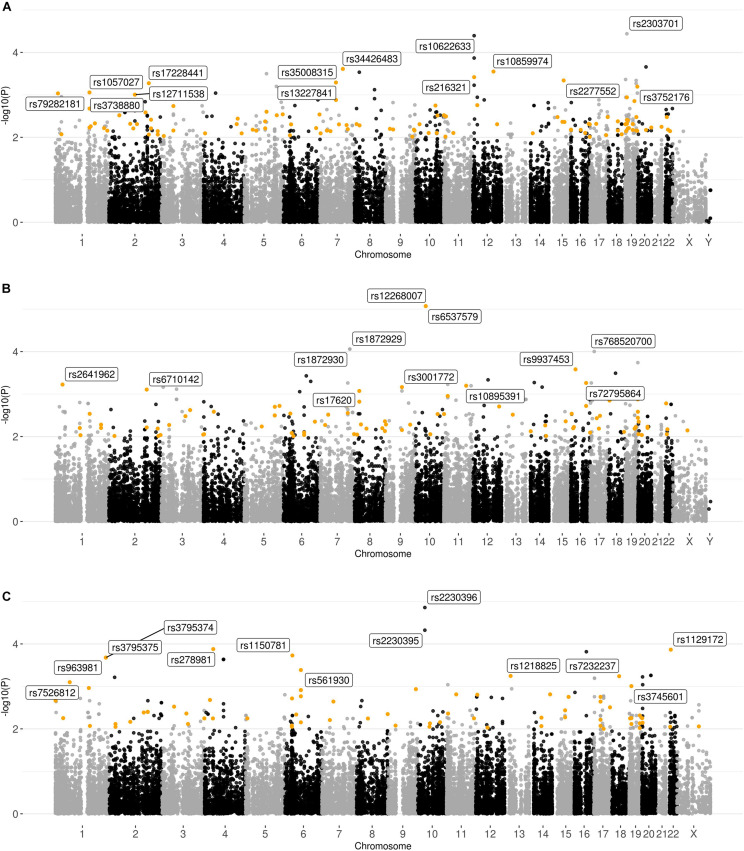
Graphical summary of the results of the association analysis of the ethnic-specific sets of polymorphisms. Plot of –log10 (*p*-values) of the χ2 test. **(A)** Buryats; **(B)** Slavs; and **(C)** Yakuts. Polymorphisms with *p*-value < 10^–2^ and high or moderate impact based on the VEP results located in the genes expressed in the testis (>1NX) are highlighted.

Based on the results of genotyping and quality analysis, 34,333, 34,770, and 34,018 polymorphisms were identified for the Buryats, Slavs, and Yakuts populations. The resulting variations were independently tested for association with impaired spermatogenesis using the χ^2^ test (Figure 5). Additionally, a logistic regression model with population stratification, age, and alcohol and tobacco consumption as covariates was applied to find the association of common variants with sperm quality. The results of the analysis of associations can be found in Supplementary Files 4–9.

As a result of the χ^2^ test, two signals with *p*-value < 10^–4^ (rs2303701 (*MAP2K7*) [*p*-value = 3.643 × 10^–5^; OR = 0.06593; synonymous variant] and rs10622633 (*VWF*) [*p*-value = 4.045 × 10^–5^; OR = NA; intron variant]) were found, as well as 26 signals with *p*-values in the range from 10^–3^ to 10^–4^ for the Buryats subset. It is noticeable that 12 out of 28 signals (rs35008315, rs34426483, rs10859974, rs216321, rs2277552, rs13227841, rs17228441, rs3752176, rs1057027, rs79282181, rs3738880, and rs12711538) had a high or moderate potential impact on the final protein product and were located in genes expressed in the tissues of the male reproductive system. One of these signals (rs17228441) is located in *FSIP2*. Previously, Martinez et al. showed the associations of mutations in *FSIP2* with MMAF (Martinez et al., 2018). Among the non-synonymous SNPs located in genes expressed in the tissues of the male reproductive system, rs35008315 and rs34426483 (*CDHR3*) showed the highest association. These homozygous genotypes for these SNPs were absent in the control sample. Using the logistic regression method did not reveal an association of SNPs located in the *CDHR3* gene. However, SNPs located in the *FSIP2* and *CDCC38* genes were among the polymorphisms with the highest association with semen quality.

In the set corresponding to the Yakut population, 33 signals with *p*-value < 10^–3^ were found, and two of them had *p*-value < 10^–4^ (rs2230396 [*p*-value = 1.389 × 10^–5^; OR = 9.50000], rs2230395) and were synonymous variants located in the same gene *ITGB1*. Moreover, among these polymorphisms, 12 have a high or moderate impact and are also localized in genes expressed in the tissues of the male reproductive system. One of these polymorphisms is rs1129172 located in the *PRAME* gene. The association of this SNP with sperm quality was also identified in the joint sample. It should be noted that after filtering polymorphisms based on their impact and genes in which they are located, all signals with *p*-value < 10^–4^ were removed as synonymous variants from the sets corresponding to the Buryats and Yakut populations. The logistic regression model confirmed the association of SNPs located in the *RBM47* and *PRAME* genes. Also, several new variations associated with sperm quality were revealed, but most of them are located in genes with low expression in the tissues of the male reproductive system. However, the non-synonymous polymorphism rs2228579 is located in the *SCNN1D* gene with significantly elevated expression in early spermatids. Moreover, the association rs557806 (*PPP1R15A*), identified in the joint sample, was also observed in the Yakut group.

However, in the case of the Slavic population, two out of five signals with *p*-value < 10^–4^ (rs12268007, rs6537579 [*p*-value = 8.482 × 10^–6^, OR = 0.0]) localized in the same gene (*WDFY4*) passed this filter. Moreover, 12 out of 31 signals with *p*-value in the range from 10^–3^ to 10^–4^ also met the conditions of this filter. As in the other groups, there was an overlap in the top lists obtained by the two methods. In particular, the logistic regression method confirmed a high level of association of polymorphisms: rs9937453, rs10895391, and rs566655 located in the *DYNC2H1*, *VWA3A*, and *LAMA1* genes, respectively. However, this method did not identify the association of SNPs located in the *WDFY4* gene. Interestingly, among the polymorphisms of the logistic regression model, SNPs associated with impaired spermatogenesis were found. Two SNPs were located in the *SUN1* gene: rs74742245 and rs59910530, and a polymorphism not represented in the dbSNP database, located at 7895446 bp on chromosome 17 in the *CHD3* gene. Both genes are expressed in the tissues of the male reproductive system. While *CHD3* exhibits low cellular specificity, the *SUN1* gene has significantly increased expression in early and late spermatids.

As in the case of the joint set, genes harboring polymorphisms obtained during the genotyping step were matched to genes associated with male infertility from the DisGeNET database. Only one signal rs3744405 (*p*-value = 7.778 × 10^–3^; OR = 3.855), which is an intron variant located in *YBX2*, was identified in the Slavs set. This gene has an extremely high expression level in testis. No other genes harboring the signals with *p*-value < 10^–3^ were found (Supplementary Files 4–9).

The data obtained was compared with the results of the association analysis for the joint set. As a result, the nine potential SNP markers rs6971091, rs557806, rs610308, rs556052, rs1289658, rs278981, rs1129172, rs12268007, and rs17228441 were selected for further analysis. The obtained variants belong to seven genes *FAM71F1*, *PPP1R15A*, *TRIM45*, *PRAME*, *RBM47*, *WDFY4*, and *FSIP2*, which played an important role in cell proliferation, meiosis, and apoptosis. All of these genes have been found to be expressed in tissues of the male reproductive system.

#### Rare Variant Association Analysis for the Joint Set

The methods used in the rare variant analysis are very sensitive to the size of the sample under study and vastly underpowered on a small sample size (Lee et al., 2014). For this reason, the analysis of rare mutations (1% < MAF < 5%) was carried out only for the joint sample (Supplementary File 10). Rare variant analysis included grouping of 32,725 variants by 13,283 genes. As expected, none of the associations passed the Bonferroni-corrected significance threshold of 0.05/13,283 = 3.8 × 10^–6^. In the joint sample, the gene with the lowest *p*-value was *ASB8* (*p*-value = 4.8 × 10^–4^).

## Discussion

Whole-exome sequencing analysis is a promising approach to identify genetic polymorphisms and genes associated with impaired spermatogenesis. We present here the first Russian WES study for identification of new polymorphisms associated with semen quality. As a result of comparing the top genetic variations obtained at the analysis of association stage, significant differences were observed between the four studied sets of samples. Thus, our results obtained were characterized by two peculiarities. First, based on WES and subsequent exome data processing, we were able to predict 10 potential SNP markers of seven genes: *FAM71F1*, *PPP1R15A*, *TRIM45*, *PRAME*, *RBM47*, *WDFY4*, and *FSIP2*, which were associated with the main features of sperm quality. Second, using analysis (Zar, 1984) of three-dimensional 3 × 3 × 2 contingency tables (3 genotypes for autosomal SNP-marker × 3 ethnic groups × 2 ranks of sperm quality), two gene groups were identified. One group included three genes *FAM71F1*, *PPP1R15A*, and *TRIM45*. For these three genes, there was no interaction between ethnicity and the other two factors—SNP genotypes and semen quality (Table 2). Therefore, the phenotypic effects of these genes were characteristic for the entire WES sample. Another four genes *PRAME*, *RBM47*, *WDFY4*, and *FSIP2* demonstrated ethnic-specific genetic effects on sperm quality. Two top genes *FAM71F1* and *PPP1R15A* from the joint set, as well as *TEX11*, were analyzed in more detail because of their considerable general genetic effects.

**FIGURE 6:**
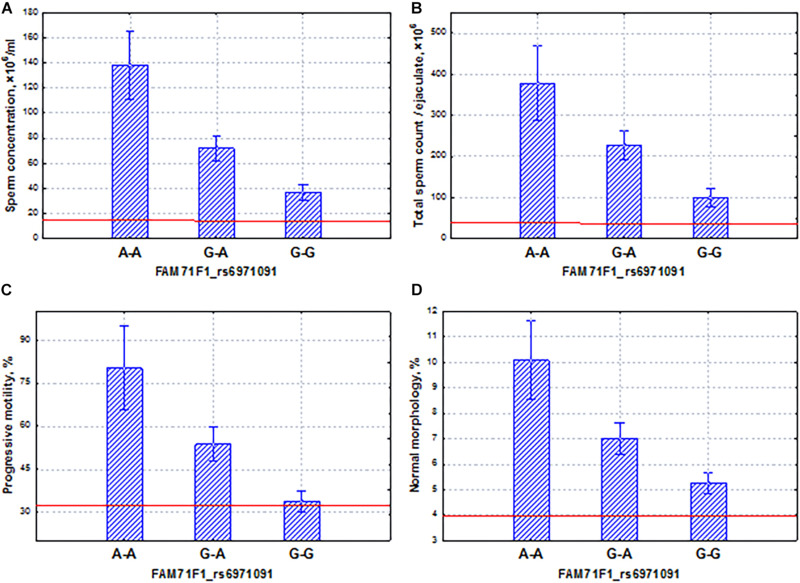
Coordinated allelic effects of the SNP marker rs6971091 of the *FAM71F1* gene on semen quality parameters. **(A)** Sperm concentration, **(B)** total sperm count, and **(C)** proportion of motile, and **(D)** morphologically normal sperm. All the allelic differences were highly significant (*p* < 0.0025). The red lines indicate the WHO reference limits for different normal semen parameters [World Health Organization (WHO), 2010].

Gene *FAM71F1* was characterized by extremely high testicular expression (Fagerberg et al., 2014). The comparative analysis of gene expression profiles in the infertile and control groups resulted in the selection of 4,946 differentially expressed genes. *FAM71F1* was included in a group of seven genes which were the most significantly downregulated genes in infertile patients (Malcher et al., 2013). In our study, we established the significant association of the SNP marker (rs6971091) of this gene with semen quality (Table 3) and identified coordinated and additive allelic effects of this marker on semen quality: the total sperm count, sperm concentration, and proportion of motile and morphologically normal spermatozoa (Figure 6). It turned out that the homozygous allele (G–G) which determines lower sperm quality in comparison with the allele (A–A) was characterized by the highest frequency in the entire WES sample. It should be noted that the alternative allele (A–A) was associated with higher values of sperm parameters but has a lower population frequency. It means that we have discovered a genetic variation associated with high activity of spermatogenesis. Moreover, all sperm characteristics of allelic variants were within the normal range according to the WHO reference values [World Health Organization (WHO), 2010]. Interestingly, extra-testicular effects have also been shown for the SNP marker (rs6971091) of this gene, in particular on obesity (Wilk et al., 2008; Gupta et al., 2012; Zlatohlavek et al., 2018).

**TABLE 3:** Polymorphisms obtained as a result of the association analysis (*p*-value < 10^–3^) of the joint set using the χ2 test with high or moderate impact based on VEP results and localized in the genes expressed in testes (>1NX).

CHR	SNP	A1	A2	F_A	F_U	P	OR	Gene	Normalized expression (NX)				
									Testis	Spermatocytes	Spermatogonia	Early spermatids	Late spermatids
chr7	rs6971091	A	G	0.1053	0.2823	5.495 × 10^–5^	0.2992	FAM71F1	64.8	11.7	5.5	1001.4	1429
chr19	rs557806	C	G	0.2947	0.1129	1.542 × 10^–4^	3.284	PPP1R15A	14.5	4.9	11.1	3.9	3.6
chr17	rs17826219	A	G	0.07979	0.2177	4.762 × 10^–4^	0.3115	ATAD5	4	32.2	35.1	12.9	4.6
chr19	rs556052	C	G	0.3789	0.1935	4.908 × 10^–4^	2.542	PPP1R15A	14.5	4.9	11.1	3.9	3.6
chr19	rs610308	G	A	0.3842	0.2016	6.412 × 10^–4^	2.471	PPP1R15A	14.5	4.9	11.1	3.9	3.6
chr1	rs1289658	G	A	0.3053	0.4919	8.59 × 10^–4^	0.4538	TRIM45	5.4	6.3	4.3	4.1	1.9
chr17	rs3764421	C	A	0.07692	0.2083	8.612 × 10^–4^	0.3167	ATAD5	4	32.2	35.1	12.9	4.6
chr17	rs11657270	C	T	0.07979	0.2097	8.897 × 10^–4^	0.3268	ATAD5	4	32.2	35.1	12.9	4.6
chr17	rs3816780	T	C	0.08065	0.2097	1.019 × 10^–3^	0.3306	ATAD5	4	32.2	35.1	12.9	4.6

*A1, minor allele; A2, major allele; F_A, frequency of the minor allele in cases; F_U, frequency of the minor allele in controls; P, p-value; NX, consensus normalized expression values from the Protein Expression Atlas.*

**FIGURE 7:**
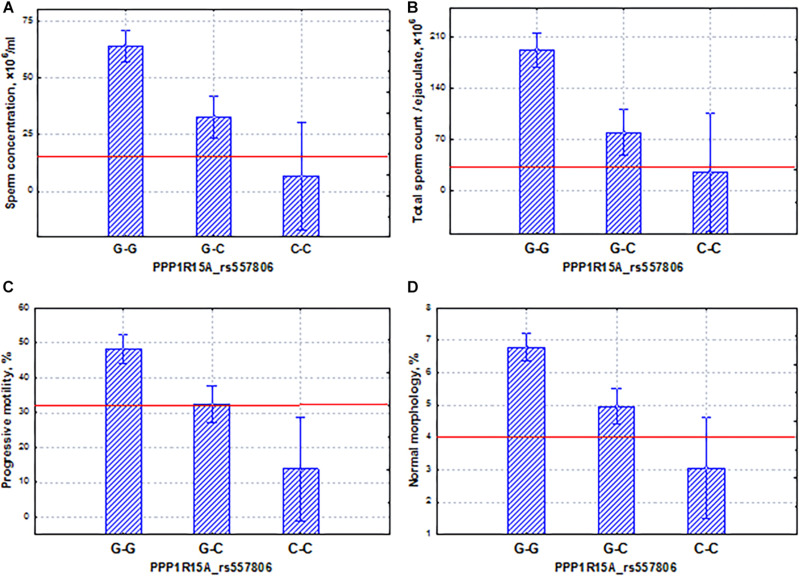
Coordinated allelic effects of the SNP marker rs557806 of the *PPP1R15A* gene on semen quality parameters. **(A)** Sperm concentration, **(B)** total sperm count, and **(C)** proportion of motile, and **(D)** morphologically normal sperm. All the allelic differences were highly significant (*p* < 0.005). The red lines indicate the WHO reference limits for different normal semen parameters [World Health Organization (WHO), 2010].

The *PPP1R15A* gene mediated cell growth arrest and apoptosis in response to DNA damage (Wu et al., 2002). It has shown its induced effect on autophagy through the suppression of the mTOR pathway during starvation (Uddin et al., 2011; Gambardella et al., 2020). We did not find any effects of this gene on testicular function in the literature. However, we found that the potential SNP marker rs557806 of the *PPP1R15A* gene has properties opposite to those of the *FAM71F1* gene. Despite the fact that this gene had a significant association with the spermatogenic pattern (Table 3), as well as coordinated and additive effects on sperm quality indicators (Figure 7), the homozygous allele (C–C), which determined low levels of spermatogenic indicators (oligo-astheno-teratozoospermia), was characterized by almost an order of magnitude lower frequency compared to the allele (G–G) of the *FAM71F1* gene (Figure 6). In other words, for this gene, the association with pathozoospermia was due to a rare variant of the SNP marker allele. Our data indicated that SNP mutations of this gene could lead to severe pathozoospermia.

**FIGURE 8:**
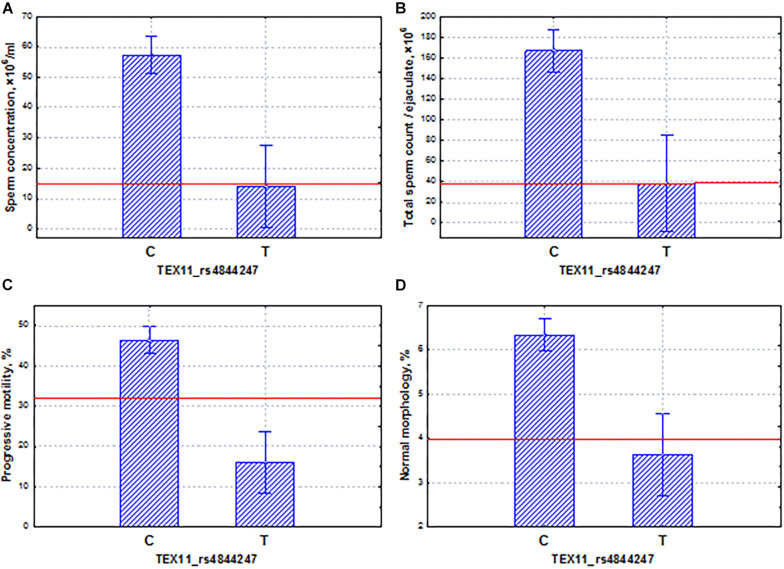
Coordinated allelic effects of the SNP marker rs4844247 of the *TEX11* gene on semen quality parameters. **(A)** Sperm concentration, **(B)** total sperm count, and **(C)** proportion of motile, and **(D)** morphologically normal sperm. All the allelic differences were highly significant (*p* < 0.005). The red lines indicate the WHO reference limits for different normal semen parameters [World Health Organization (WHO), 2010].

Another interesting example is the X-linked *TEX11* gene, which is expressed only in male germ cells, mainly in spermatogonia. This gene is involved in the organization of the synaptonemal complex, and its mutations can lead to the arrest of meiosis and the development of azoospermia (Yang et al., 2015; Yatsenko et al., 2015; Nakamura et al., 2017; Sha et al., 2018; Tüttelmann et al., 2018; Cannarella et al., 2021). In this study, we found that the phenotypic effect of the *TEX11* gene on spermatogenic function was similar to that of the *PPP1R15A* gene. The *TEX11* gene showed the significant association with the spermatogenic pattern and the coordinated effects on the main indicators of sperm quality (Figure 8). The allele (T) of the *TEX11* gene led to the development of oligoasthenoteratozoospermia, a severe disorder of spermatogenesis. On average, the carriers of this mutation had a decrease in the total sperm count, sperm concentration, and proportion of motile and morphologically normal sperm (Figure 8) below the reference values for normal ejaculate quality (World Health Organization (WHO), 2010). It appeared that the allelic frequency in the pathozoospermia group was more seven times higher than in the normospermia group (Supplementary File 1).

**FIGURE 9:**
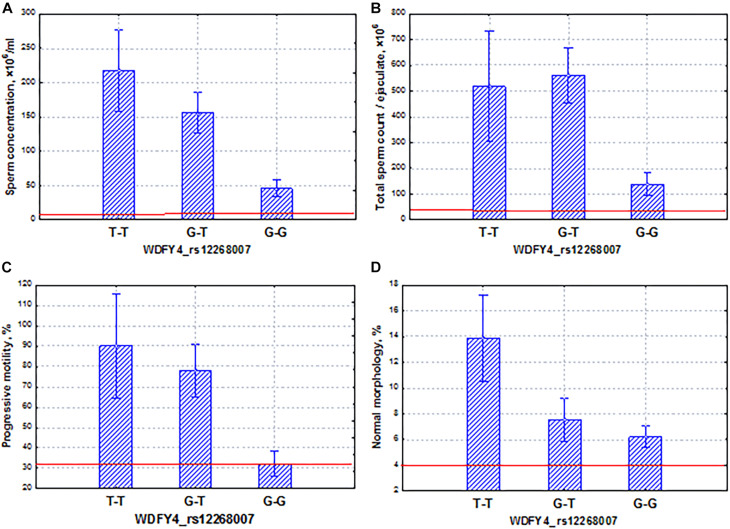
Coordinated allelic effects of the SNP marker rs12268007 of the *WDFY4* gene on semen quality parameters. **(A)** Sperm concentration, **(B)** total sperm count, and **(C)** proportion of motile, and **(D)** morphologically normal sperm. All the allelic differences were highly significant (*p* < 0.005). The red lines indicate the WHO reference limits for different normal semen parameters [World Health Organization (WHO), 2010].

Among the ethno-specific associations with a *p*-value < 10^––4^, the *WDFY4* gene in the Slavic population should be considered in more detail. This gene plays an important role in autophagic biological processes and may highly express in early and late spermatids^2^. Genetic variants in and around the *WDFY4* gene were identified as associated with systemic lupus erythematosus (Yang et al., 2010) and with severe tick-borne encephalitis (Ignatieva et al., 2019). In our study, we found that the SNP marker rs12268007 of the *WDFY4* gene had the same properties as the *FAM71F1* gene. Specifically, we established the coordinated and additive allelic effects of this marker on sperm quality: sperm concentration, total sperm count, and proportion of motile and morphologically normal spermatozoa (Figure 9). Similar to the *FAM71F1* gene, the homozygous allele (G–G) determined lower sperm quality in comparison with the allele (T–T) and additionally was characterized by the highest frequency in the Slavic population. In contrast, the alternative allele (T–T) was associated with higher values of sperm parameters but had a lower population frequency. It is worth mentioning that all sperm characteristics of allelic variants were within the normal range according to the WHO reference values [World Health Organization (WHO), 2010]. Using the example of these two genes (*FAM71F1* and *WDFY4*), we are able to demonstrate a genetic variation associated with high activity of spermatogenesis.

**FIGURE 10:**
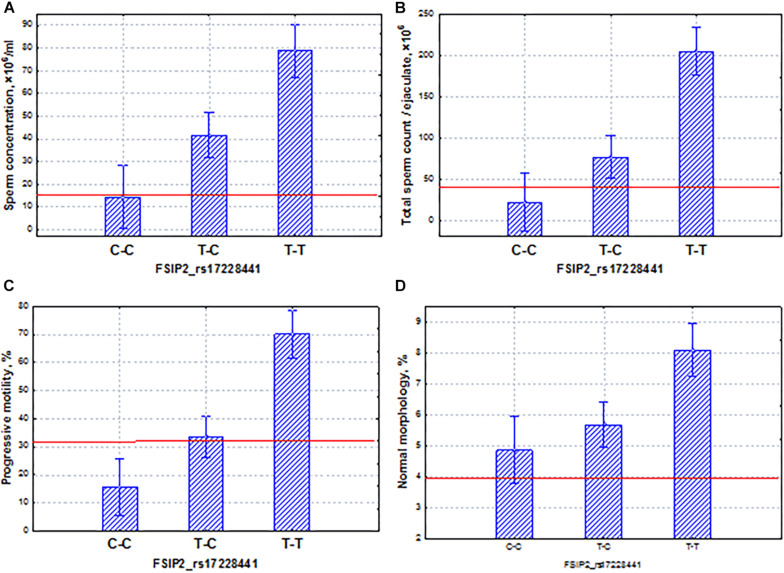
Coordinated allelic effects of the SNP marker rs17228441 of the *FSIP2* gene on semen quality parameters. **(A)** Sperm concentration, **(B)** total sperm count, and **(C)** proportion of motile, and **(D)** morphologically normal sperm. All the allelic differences were highly significant (*p* < 0.005). The red lines indicate the WHO reference limits for different normal semen parameters [World Health Organization (WHO), 2010].

The *FSIP2* gene demonstrated associations with sperm quality at a *p*-value < 5 × 10^–4^ in the Buryats population. This gene encodes a protein associated with the sperm FS. Genes encoding most of the fibrous-sheath-associated protein genes are transcribed only during the post-meiotic period of spermatogenesis. The protein encoded by this gene is specific to spermatogenic cells. Mutations in *FSIP2* were associated with a complete disorganization of the FS and axonemal defects (Martinez et al., 2018; NsotaMbango et al., 2019). In our case, the SNP marker rs17228441 of the *FSIP2* gene had a coordinated and additive effect on sperm quality indicators (Figure 10). In particular, the homozygous allele (C–C) determined the low level of all semen parameters (oligoasthenoteratozoospermia); the allele (T–T) was characterized by opposite properties. Our data showed that SNP mutations of this gene could lead to severe pathozoospermia.

**FIGURE 11:**
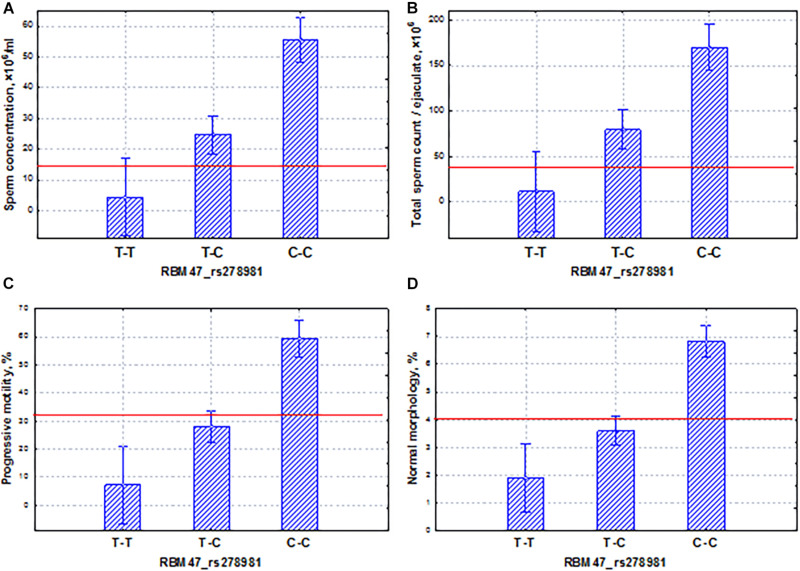
Coordinated allelic effects of the SNP marker rs278981 of the *RBM47* gene on semen quality parameters. **(A)** Sperm concentration, **(B)** total sperm count, and **(C)** proportion of motile, and **(D)** morphologically normal sperm. All the allelic differences were highly significant (*p* < 0.005). The red lines indicate the WHO reference limits for different normal semen parameters [World Health Organization (WHO), 2010].

In the Yakut population, it was worth choosing two genes that had different features: allelic variants of one gene (*RBM47*) could lead to pathozoospermia, and the other (*PRAME*) could induce variability in the normospermia region. The *RBM47* gene has controlled several aspects of RNA biogenesis, including splicing, localization, stability, and translation efficiency. RBM47 represented a novel molecular switch of cell fate decisions that functions as a regulator of the p53/p21-signaling axis (Radine et al., 2020). In our study, the homozygous allele (T–T) of the *RBM47* gene induced oligoasthenoteratozoospermia, whereas the allele (T–T) determined high levels of all sperm traits. The additive inheritance was observed for all sperm traits: the total sperm and sperm concentration and the proportion of motile and morphologically normal spermatozoa (Figure 11).

**FIGURE 12:**
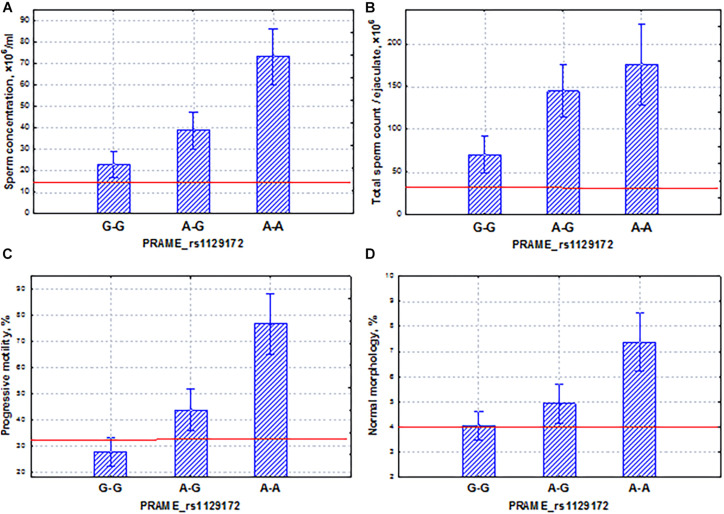
Coordinated allelic effects of the SNP marker rs1129172 of the *PRAME* gene on semen quality parameters. **(A)** Sperm concentration, **(B)** total sperm count, and **(C)** proportion of motile, and **(D)** morphologically normal sperm. All the allelic differences were highly significant (*p* < 0.05). The red lines indicate the WHO reference limits for different normal semen parameters [World Health Organization (WHO), 2010].

The second *PRAME* gene, found in the Yakut population, had the effect in the area of normospermia. The SNP marker rs1129172 of the *PRAME* gene had a coordinated and additive effect on all sperm quality indicators (Figure 12). It is known that among all cancer testis antigens, *PRAME* is in a unique position as it not only is widely expressed in various cancers but also elicits specific cellular immune responses against numerous neoplastic cells, as well as against leukemia progenitor cells, and plays a role in the innate immune response (Al-Khadairi and Decock, 2019). The *PRAME* gene is not only expressed in the normal testis but also widely expressed in numerous cancers. Moreover, PRAME can act as an oncogene or a tumor-suppressor gene in different cancer types. PRAME exerts its biological functions via regulation of its downstream targets, such as p53, p21, Bcl-2, TRAIL, RAR, Hsp27, and S100A4 in human malignancies (Xu et al., 2020). In our study, we also demonstrated that the *PRAME* gene had a modulating effect on the activity of spermatogenesis.

The main limitations of our study that should be acknowledged were sample size for WES, which reduced our statistical power, and high heterogeneity of studying phenotypes. The small size of the sample under study also affects the significance of the results of the rare variant analysis. Moreover, in this study, the main emphasis was on the search for non-synonymous variations in exon regions, while mutations in introns, as well as synonymous polymorphisms, can also have a significant impact. Despite these limitations, we revealed a set of promising SNP markers of impaired spermatogenesis and semen quality. In addition, the revealed associations need to be validated in further experiments, so they should be interpreted with caution until their validity is established by independent studies. In particular, we are going to verify the associations of selected potential SNP markers using our previously collected population (about 1,500 males). The future verification of potential SNP markers might help to create a clinical gene panel for the diagnosis of male idiopathic infertility, based on our results of WES. Summarizing the results, we can conclude that a preselected group of participants, including three ethnic groups with pathozoospermia and normospermia, allowed us to reveal a genetic variation that determines not only idiopathic spermatogenic failure but also a very high activity of spermatogenesis.

## Data Availability Statement

The sequencing data generated in this study are available from the corresponding author upon reasonable request.

## Ethics Statement

The studies involving human participants were reviewed and approved by the ethics committee of the Federal Research Center “Institute of Cytology and Genetics,” the Siberian Branch of the Russian Academy of Sciences. The patients/participants provided their written informed consent to participate in this study.

## Author Contributions

SK performed WES data analysis and association analysis. GV contributed to whole-exome sequencing. LO provided the overall supervision of the project and collected the questionnaires and samples. MK contributed to semen analysis and men sample collection. AO contributed to the concept and study design, men sample collection, and data statistical analysis. SK, LO, and AO corrected the manuscript. All authors have read and approved the final manuscript.

## Conflict of Interest

The authors declare that the research was conducted in the absence of any commercial or financial relationships that could be construed as a potential conflict of interest.

## Footnotes

1
www.r-project.org

2
https://www.proteinatlas.org/ENSG00000128815-WDFY4/celltype

## Supplementary Material

Supplementary File 1 Meta information about the studied sample.

Supplementary File 2 Results of association analysis using χ2 test for the whole sample combined with VEP results, Protein Expression Atlas normalized expression values for genes harboring polymorphisms and genes associated with male infertility from DisGeNET.

Supplementary File 3 Top-100 results of association analysis using log-regression model for the whole sample combined with VEP results, Protein Expression Atlas normalized expression values for genes harboring polymorphisms and genes associated with male infertility from DisGeNET.

Supplementary File 4 Results of association analysis using χ2 test for the Buryat subgroup combined with VEP results, Protein Expression Atlas normalized expression values for genes harboring polymorphisms and genes associated with male infertility from DisGeNET.

Supplementary File 5 Results of association analysis using χ2 test for the Slavic subgroup combined with VEP results, Protein Expression Atlas normalized expression values for genes harboring polymorphisms and genes associated with male infertility from DisGeNET.

Supplementary File 6 Results of association analysis using χ2 test for the Yakut subgroup combined with VEP results, Protein Expression Atlas normalized expression values for genes harboring polymorphisms and genes associated with male infertility from DisGeNET.

Supplementary File 7 Top-100 results of association analysis using log-regression model for the Buryat subgroup combined with VEP results, Protein Expression Atlas normalized expression values for genes harboring polymorphisms and genes associated with male infertility from DisGeNET.

Supplementary File 8 Top-100 results of association analysis using log-regression model or the Slavic subgroup combined with VEP results, Protein Expression Atlas normalized expression values for genes harboring polymorphisms and genes associated with male infertility from DisGeNET.

Supplementary File 9 Top-100 results of association analysis using log-regression model for the Yakut subgroup combined with VEP results, Protein Expression Atlas normalized expression values for genes harboring polymorphisms and genes associated with male infertility from DisGeNET.

Supplementary File 10 Results of rare-variant analysis for the whole sample using SKAT.
