# Correction: Whole-exome sequencing analysis of human semen quality in Russian multiethnic population

**DOI:** 10.3389/fgene.2026.1837698

**Published:** 2026-04-09

**Authors:** Semyon Kolmykov, Gennady Vasiliev, Ludmila Osadchuk, Maxim Kleschev, Alexander Osadchuk

**Affiliations:** 1 Institute of Cytology and Genetics, Siberian Branch of Russian Academy of Sciences, Novosibirsk, Russia; 2 Department of Computational Biology, Sirius University of Science and Technology, Sochi, Russia

**Keywords:** whole-exome sequencing, association analysis, semen quality, pathozoospermia, normospermia, Slavs, Buryats, Yakuts

The Data Availability statement was erroneously given as “The original contributions presented in the study are publicly available. This data can be found here: https://www.ncbi.nlm.nih.gov/bioproject/PRJNA733014.”

Due to legal requirements related to personal genomic data protection in the Russian Federation, the data are being withdrawn from public repositories.

The correct Data Availability statement is “The sequencing data generated in this study are available from the corresponding author upon reasonable request.”

The original article has been updated.

