# Supplementary material for: Whole-Exome Sequencing Analysis of Human Semen Quality in Russian Multiethnic Population
Source: Front Genet. 2021 Jun 11;12:662846. doi: 10.3389/fgene.2021.662846 (PMC8232892; doi:10.3389/fgene.2021.662846)
Supplement: Supplementary file 11 [file Presentation_1.pdf]

frontiers

Supplementary Material

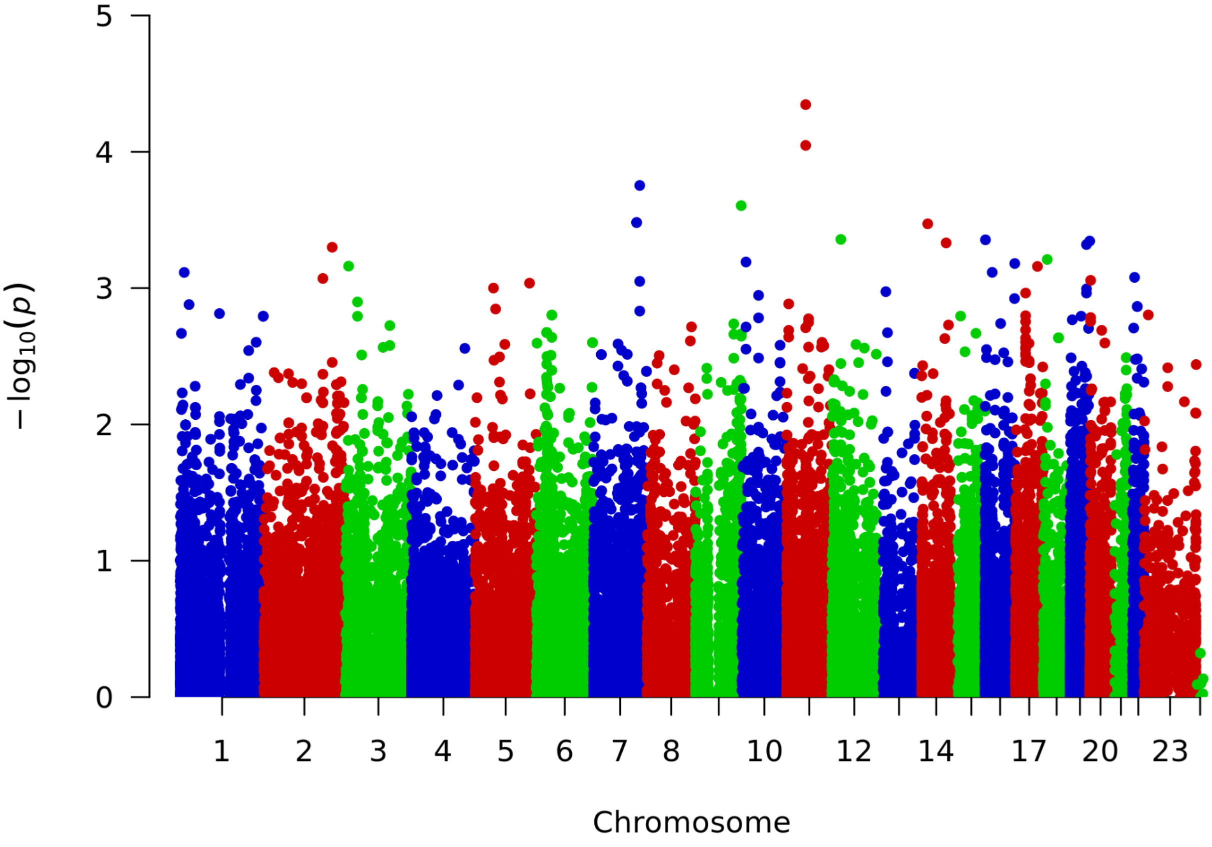

Supplementary Figure 1. Graphical summary of the results of the association analysis of 157 samples. Plot of -log10(P-values) of the log-regression model.

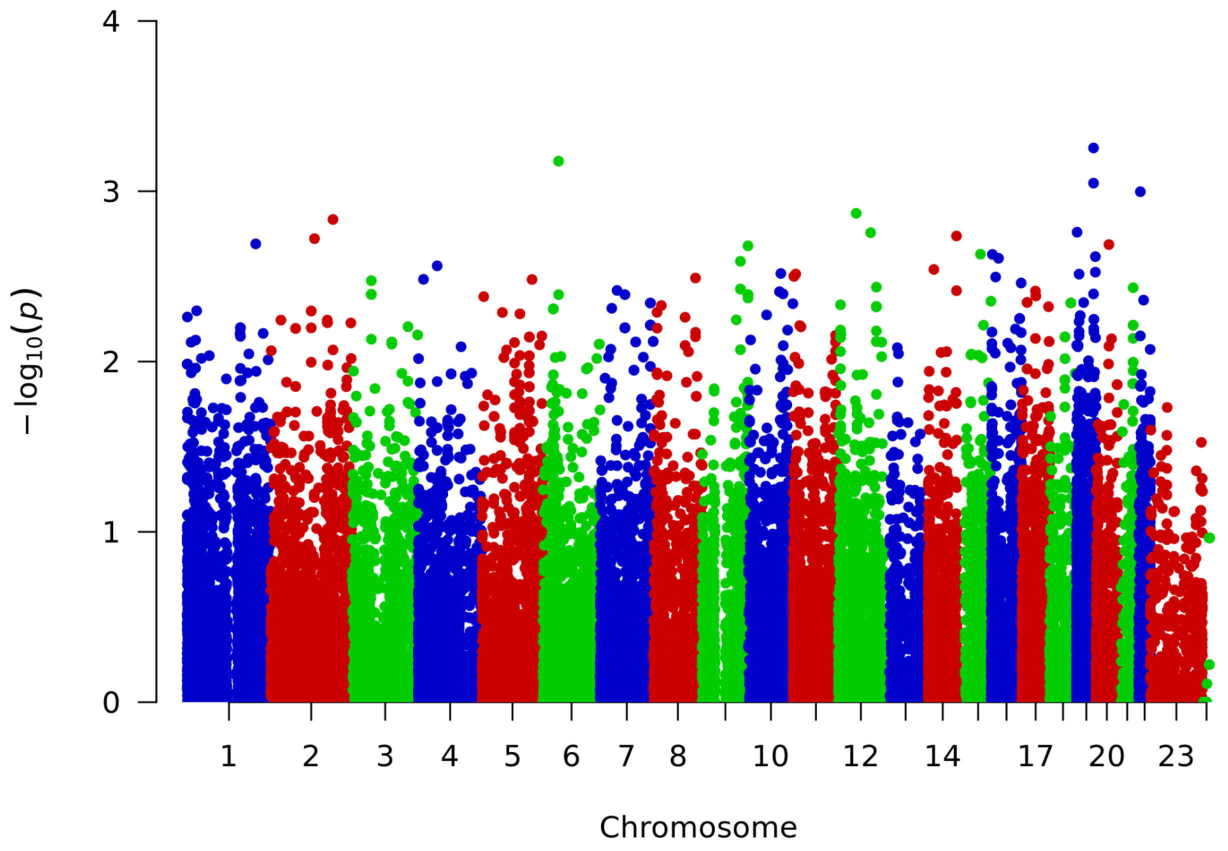

Supplementary Figure 2. Graphical summary of the results of the association analysis of the Buryats group. Plot of -log10(P-values) of the log-regression model.

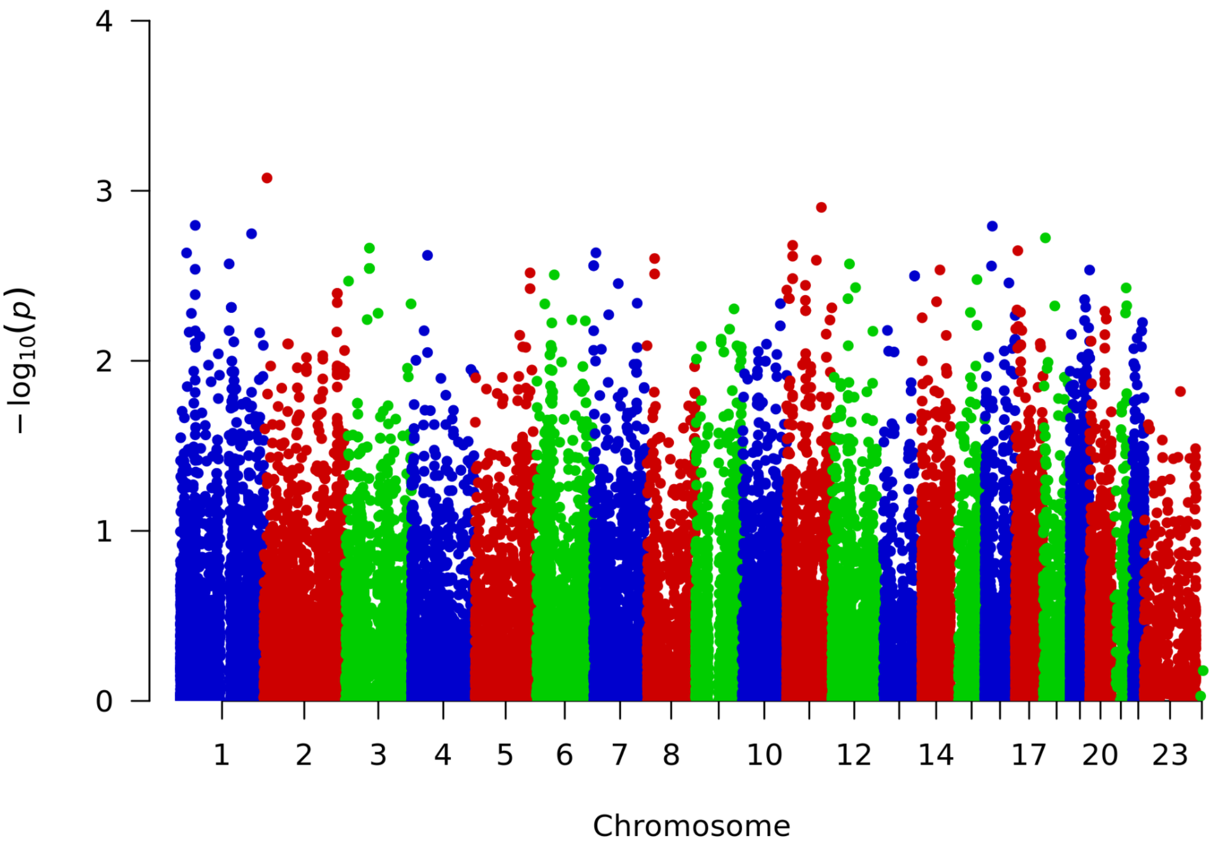

Supplementary Figure 3. Graphical summary of the results of the association analysis of the Slavs group. Plot of -log10(P-values) of the log-regression model.

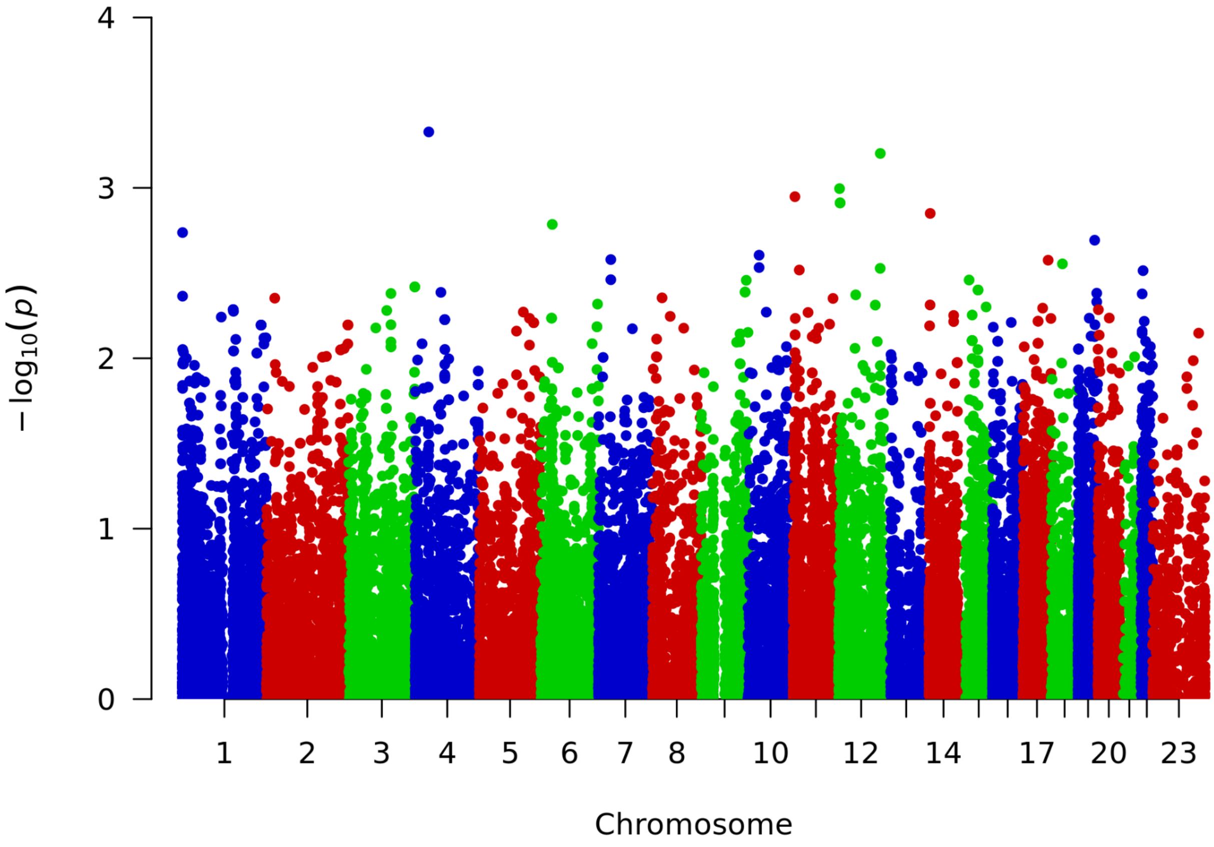

Supplementary Figure 4. Graphical summary of the results of the association analysis of the Yakuts group. Plot of -log10(P-values) of the log-regression model.
